# Environmental factors and host genotype control foliar epiphytic microbial community of wild soybeans across China

**DOI:** 10.3389/fmicb.2023.1065302

**Published:** 2023-03-13

**Authors:** Rui Zhou, Gui-Lan Duan, Pablo García-Palacios, Guang Yang, Hui-Ling Cui, Ming Yan, Yue Yin, Xing-Yun Yi, Lv Li, Manuel Delgado-Baquerizo, Yong-Guan Zhu

**Affiliations:** ^1^State Key Laboratory of Urban and Regional Ecology, Research Center for Eco-Environmental Sciences, Chinese Academy of Sciences, Beijing, China; ^2^University of Chinese Academy of Sciences, Beijing, China; ^3^Instituto de Ciencias Agrarias, Consejo Superior de Investigaciones Científicas, Madrid, Spain; ^4^Laboratorio de Biodiversidad y Funcionamiento Ecosistémico, Instituto de Recursos Naturales y Agrobiología de Sevilla (IRNAS), CSIC, Sevilla, Spain; ^5^Unidad Asociada CSIC-UPO (BioFun), Universidad Pablo de Olavide, Sevilla, Spain; ^6^Institute of Urban Environment, Chinese Academy of Sciences, Xiamen, China

**Keywords:** wild soybean, foliar microbiome, microbial community assembly, core microbiome, host genotype

## Abstract

**Introduction:**

The microbiome inhabiting plant leaves is critical for plant health and productivity. Wild soybean (*Glycine soja*), which originated in China, is the progenitor of cultivated soybean (*Glycine max*). So far, the community structure and assembly mechanism of phyllosphere microbial community on *G. soja* were poorly understood.

**Methods:**

Here, we combined a national-scale survey with high-throughput sequencing and microsatellite data to evaluate the contribution of host genotype vs. climate in explaining the foliar microbiome of *G. soja*, and the core foliar microbiota of *G. soja* were identified.

**Results:**

Our findings revealed that both the host genotype and environmental factors (i.e., geographic location and climatic conditions) were important factors regulating foliar community assembly of *G. soja*. Host genotypes explained 0.4% and 3.6% variations of the foliar bacterial and fungal community composition, respectively, while environmental factors explained 25.8% and 19.9% variations, respectively. We further identified a core microbiome thriving on the foliage of all *G. soja* populations, including bacterial (dominated by *Methylobacterium*-*Methylorubrum*, *Pantoea*, *Quadrisphaera*, *Pseudomonas*, and *Sphingomonas*) and fungal (dominated by *Cladosporium*, *Alternaria*, and *Penicillium*) taxa.

**Conclusion:**

Our study revealed the significant role of host genetic distance as a driver of the foliar microbiome of the wild progenitor of soya, as well as the effects of climatic changes on foliar microbiomes. These findings would increase our knowledge of assembly mechanisms in the phyllosphere of wild soybeans and suggest the potential to manage the phyllosphere of soya plantations by plant breeding and selecting specific genotypes under climate change.

## 1. Introduction

Microbial communities that colonize and thrive on the surfaces of leaves include bacteria, fungi, viruses, cyanobacteria, actinomycetes, nematodes, and protozoans (Bashir et al., 2022). These foliar microorganisms perform significant roles in regulating the development and health of plants, including nutrient acquisition, disease resistance, and stress tolerance (Bashir et al., 2022). However, unlike other plant compartments that have been traditionally studied in agricultural research, such as root endosphere and rhizosphere (Bay et al., 2021; Zheng et al., 2021), the biotic and abiotic factors that contribute to the assembly of the foliar microbiome as well as the composition of the core foliar microbiome, remain far from understanding.

In general, the foliar microbiome is considered to be regulated by multiple factors, including climate and host genetic diversity. First, environmental perturbations, including global change, play an important role in shaping phyllosphere microbial communities (Zhu et al., 2022). For instance, warmer temperatures and increased precipitation are anticipated to increase the proportion of potential plant pathogens in the phyllosphere (Aydogan et al., 2018; Chen et al., 2021). In addition, previous studies have demonstrated that the same genotype sampled across different sites has significantly distinct phyllosphere microbial composition, diversity, and structure (Xing et al., 2021; Abdelfattah et al., 2021a), indicating that environmental conditions play a significant role in determining phyllosphere microbial communities. Moreover, according to some previous studies, the host genotype is a significant factor that drives the phyllosphere microbial community assembly of model plants under controlled conditions (Sapkota et al., 2015; Li Y. et al., 2021; Shakir et al., 2021) and the various microbial taxa present in different cultivars have a significant impact on plant health (Wang et al., 2021). However, there is still significant uncertainty on how genetic distance controls the foliar microbiome in plants that are growing under changing climates. To design efficient plant breeding programs and farming practices promoting plant health, it is essential to quantify the contribution of genetic distance relative to the environmental filter in driving the foliar microbiome. Furthermore, we need to advance our understanding of the composition of the core plant microbiome. The core microbiome is considered as the microbial taxa that constitute among the majority of samples from a particular host or environment (Yeoh et al., 2017; Neu et al., 2021; Abdelfattah et al., 2022), and has been demonstrated to exhibit beneficial effects on biological nitrogen fixation, disease suppression, and host growth promotion (Taye et al., 2020; Zhang et al., 2022). Thus, the core microbiome of several crops has been identified in the rhizosphere (Walters et al., 2018; Xu et al., 2018) and endosphere (Kumar et al., 2021). However, the core microbiome of the crop’s foliar microbiome is rarely addressed.

In this study, we analyzed the foliar microbiomes of wild soybean across eight sites distributed throughout China to elucidate the fundamental biotic and abiotic drivers. Soybean (*Glycine max*), which is rich in vegetable protein and oil, is one of the most important crops in the world, with a global production of 2,784 hg/ha in 2020. Since ancient times, soybean has been a staple in Chinese cuisine, and presently it is also processed into animal feed to meet the protein demands of modern livestock production in China. Wild soybean (*Glycine soja*), the progenitor of soybean, which originated in China, has been listed as a national second-class protected plant since 1999 (He et al., 2012). It has been demonstrated that the wild progenitor could primordially reflect the composition of host-associated microbes (Morvan et al., 2020). However, for cultivated varieties, the microbial diversity and structure could be altered during domestication as a result of microbiome introgression or loss (Ma et al., 2019; Abdelfattah et al., 2022). Therefore, wild soybean is the optimal material to investigate soybean-associated microbes. Owing to complex geographic and ecological conditions, there are many genotypes of wild soybean distributed throughout China following long-term climatic and environmental selection (Guo et al., 2012; Li and Wang, 2020). Current studies have concentrated on the microbiome of root nodules (Yang et al., 2020; Zheng et al., 2020) and the rhizosphere (Chang et al., 2019; Tian et al., 2020) to investigate the microbial nitrogen fixation capacity in this legume species (Mahmud et al., 2020). However, little information has been reported on the importance of climate and host genotype in regulating the foliar microbiome of wild soybeans. Moreover, the core leaf microbiome of wild soybean is still unexplored.

Therefore, the objectives of this study were to: (1) characterize the foliar microbial community composition and diversity of the wild soybeans under various host genotypes and growth conditions; (2) analyze the variable importance of the host genotype and environmental factors in foliar community assembly; (3) identify the core foliar microbes of the wild soybeans as well as the factors driving their assembly. According to our hypothesis, the foliar microbial community is significantly influenced by both climate and host genotype, and the core microbes could be beneficial to plant growth. The genetic distance is an effective tool for understanding foliar epiphytic microbial variation, which could pave the way for the promotion of certain microbiomes by modifying genetic variability.

## 2. Materials and methods

### 2.1. Study site and sampling

The foliage and seeds of *G. soja* were collected during the flowering stage in 2019 from eight major wild soybean distribution locations in China, across the north to south (26.31°N to 40.45°N, 115.95°S to 117.40°S) (Supplementary Figure S1A; Supplementary Table S1). As a result of significant climate changes between the north and south sides of the Qinling Mountains-Huaihe River Line in China, the sampling sites were divided into northern and southern regions (Fang et al., 2002). In the northern regions, samples were obtained from wild soybean from Yanqing country and Dongjiao Park in Beijing City, Dahuangbao Park in Tianjin City, Tangshan city from Hebei Province, Weishan country in Shandong Province, and Sanmenxia city from Henan Province. These samples were recorded as YQ, DG, DHB, CFD, WS, and SMX, respectively. In the southern regions, samples of wild soybean were acquired from Loudi country from Hunan Province and Mingxi country from Fujian Province. These samples were recorded as LD and GD, respectively. The two most distant sampling locations (from Yanqing to Mingxi) were more than 1900 km apart, while the two near sampling locations (from Yanqing to Dongjiao) were at least 100 km apart.

The foliage and seed samples from the wild soybean population were randomly collected at each location. Briefly, 20 to 30 leaves were collected from a single plant for a sample. Three to five plants were obtained from each location to represent different biological replicates. The rubber gloves and the pruners were sprayed with 75% ethanol and wiped each time to prevent cross-contamination. The foliage samples were stored in containers with ice bags, while the seeds were stored in envelopes at room temperature. These sample containers and envelopes were transported to the laboratory within 1–2 days, where the leaves were subsequently frozen at −20°C for further experiments. The geographic coordinates (latitude and longitude) of sampling locations were recorded, and the climatic variables, including mean annual precipitation (MAP), mean annual temperature (MAT), and global horizontal irradiance (GHI), were obtained from the Resource and Environment Science and Data Center (Supplementary Table S2).^1^

### 2.2. *G. soja* genotype identification

The genomic DNA of *G. soja* was extracted from 0.1 g leaves (wet sample) of each sample using the plant genomic DNA kit (TIANGEN, Beijing, China). The quality and concentration of DNA were assessed using the Nano-300 instrument (Allsheng, China). As the wild soybean genome exhibits considerable polymorphism, simple sequence repeat (SSR) molecular marker technology was employed to determine plant genetic variation (Yan et al., 2008). From the constructed genetic linkage map of the soybean genome (Cregan et al., 1999), 20 primer pairs were selected for polymerase chain reaction (PCR) amplification (Supplementary Table S3). Each of them was sequenced separately. The reaction mixture of PCR amplification contained 2 μl DNA template (30 ng/μL), 1 μl primer (10 mM), 5 μl mix (2 × Taq PCR StarMix), and 2 μl double-distilled water (ddH_2_O). The program comprised initial denaturing at 94°C for 4 min, followed by 30 cycles of denaturing at 94°C for 30 s, annealing at 47°C for 30 s, extension at 72°C for 30 s, and final extension at 72°C for 10 min. The amplification products were subsequently separated by polyacrylamide gel electrophoresis (PAGE) for 2 h, and the bands were visualized using silver staining. The genetic distance (Nei, 1972) was estimated using PopGene32 software (Supplementary Table S4).

Following genotype identification, the plants sampled from the same location with the same genotype (e.g., with the same SSR sequence) were selected as replicates for this location. A total of 37 samples were obtained from eight locations (Supplementary Table S1).

### 2.3. Foliar epiphytic microbial DNA extraction and amplicon sequencing

Soybean leaves weighing 2 g from each sample were deposited into a conical flask containing 40 mL of sterile phosphate-buffered saline (PBS: NaCl, KCl, Na_2_HPO_4_, and KH_2_PO_4_) with 0.1% Triton X-100 (pH 7.4) (Duran et al., 2018), and thereafter the conical flasks were subjected to sonication for 5 min and shaken (180 rpm) at 25°C for 1 h. Subsequently, the mixture was filtered, initially with a sterilized nylon gauze, followed by a 0.22 μm cellulose membrane (Yin et al., 2022). The FastDNA SPIN Kit for Soil (MP Biomedicals, Solon, USA) was employed to extract the total DNA from the resulting epiphytic fraction, and the quality of DNA obtained was assessed using NanoDrop One instrument (Nanodrop, USA). Then the DNA samples were stored at −20°C until further analysis.

For the high-throughput Illumina sequencing, the V4-V5 region of the bacterial 16S rRNA gene (16S primers 515F-907R) and the ITS1 region of the fungal ITS rRNA gene (ITS primers ITS1-ITS2) were targeted (Supplementary Table S5). The PCR program was as follows: 95°C for 3 min, followed by 30 cycles of 95°C for 30 s, 55°C for 30 s, 72°C for 45 s, and a final extension at 72°C for 10 min. Each of the 37 samples was sequenced independently. Consecutively, negative controls (in which the DNA template was replaced by sterile water) were performed to exclude contamination through PCR amplification. Using the Illumina NovaSeq PE250 platform (Majorbio, Shanghai, China), the amplicons were purified, quantified, pooled, and sequenced. All raw sequencing data were deposited in the National Center for Biotechnology Information (NCBI) Sequence Read Archive (SRA) with accession number PRJNA862265.

The QIIME 2 Pipeline was employed to process the raw sequence data (Caporaso et al., 2010). In brief, the primers, low-quality sequences (average base quality score < 25), and chimeric and barcode sequences were eliminated, and the paired-end sequences were merged to a single sequence. The table generation of amplicon sequence variants (ASVs) was performed according to DADA2 analysis (Callahan et al., 2016). A total of 2,950,325 bacterial sequences and 7,579,465 fungal sequences were obtained. Silva 16S rRNA gene database (Version 138^2^) and the UNITE fungal database (Version 8) (Nilsson et al., 2019) were used to assign bacterial and fungal taxonomy, respectively. The host DNA (chloroplast and mitochondria) sequences and archaea taxa were removed from bacterial ASVs. Low-abundance ASVs with read counts of less than five across all the samples were discarded prior to downstream analysis (Kim et al., 2020). A total of 2,058 bacterial ASVs and 4,490 fungal ASVs were produced using this process. Following normalization, 14,648 bacterial sequences and 111,063 fungal sequences were obtained per sample. The resulting sequences were employed for further analysis.

### 2.4. Statistical analyses and visualization

All statistical analyses were performed using R version 4.1.2 and SPSS version 22. Scheffe’s test for multiple comparisons was used in conjunction with a one-way analysis of variance (ANOVA) to determine statistical significance at ɑ = 0.05. If the variances of observations were heterogeneous, the nonparametric Kruskal-Wallis test (multiple groups) and Mann–Whitney U test (two groups) were employed to assess the statistical significance. Using the *diversity* and *rowSums* functions in the “vegan” package (Oksanen et al., 2022), the alpha-diversity indices, including the Shannon index and richness (observed species), were determined. Bray–Curtis distance-based NMDS ordinations at the genus level were calculated in R using the “vegan” package (function = *metaMDS*). The geographic distance matrix was generated according to the geographic coordinates of sampling sites (latitude and longitude) using the “geosphere” package (Milici et al., 2016). The Mantel test with Spearman’s correlation (999 permutations) was performed to ascertain if the dissimilarity in microbial communities was related to geographic or host genetic distance. Variation partition analysis was employed to determine the shared effects of explaining variables grouped within various categories in the “vegan” package (Li H. Y. et al., 2021). Hierarchical clustering was performed using the “vegan” package (function = *hclust*) based on the Bray-Curtis matrices. Host phylogeny was performed (function = *bionj*) based on the neighbor-joining in accordance with the host genetic distance matrices. Using “ggtree” package, both microbial hierarchical clustering and host phylogeny were visualized (Yu et al., 2017). Using the Mantel test, the correlation between the host phylogeny and microbial hierarchical clustering was determined (Spearman, 999 permutations). Core ASVs were identified in accordance with the prevalence threshold of 70% (Abdelfattah et al., 2022). Redundancy analysis (RDA) was performed using the “vegan” package, and the importance of each explanatory variable was calculated by hierarchical partitioning using “radcca.hp” package (Lai et al., 2022). Heatmaps and Venn diagrams were performed with “pheatmap” and “VennDiagram” packages, respectively. Using BLAST,^3^ the sequences of core microbial ASVs were compared with those in the NCBI GenBank, and a Neighbor-Joining tree (Saitou and Nei, 1987) was constructed in MEGA-X. The bootstrap consensus tree was inferred from 1,000 replicates, and the phylogenetic tree was visualized using iTOL.^4^

## 3. Results

### 3.1. Host phylogenetic relationships

Through SSR sequencing, we found that plants within the same location had the same genotype, and those in different locations had different genotypes. The phylogenetic tree based on microsatellite data showed that the eight wild soybean populations were statistically clustered into three major groups associated with their geographical locations(Supplementary Figure S1B). The southern populations (GD and LD), as well as the samples from WS and DG, were clustered into the same group. The samples from DHB, YQ, and SMX were clustered into one group, and the samples from CFD were segregated into another group, making two groups of the northern populations. These results demonstrated that the distribution of wild soybean genotypes is mostly related to geographical conditions, while the phylogenetic relationships of the wild soybean were different from the geographic relationships among sampling locations.

### 3.2. Composition and diversity of the foliar bacterial and fungal communities

**Figure 1:**
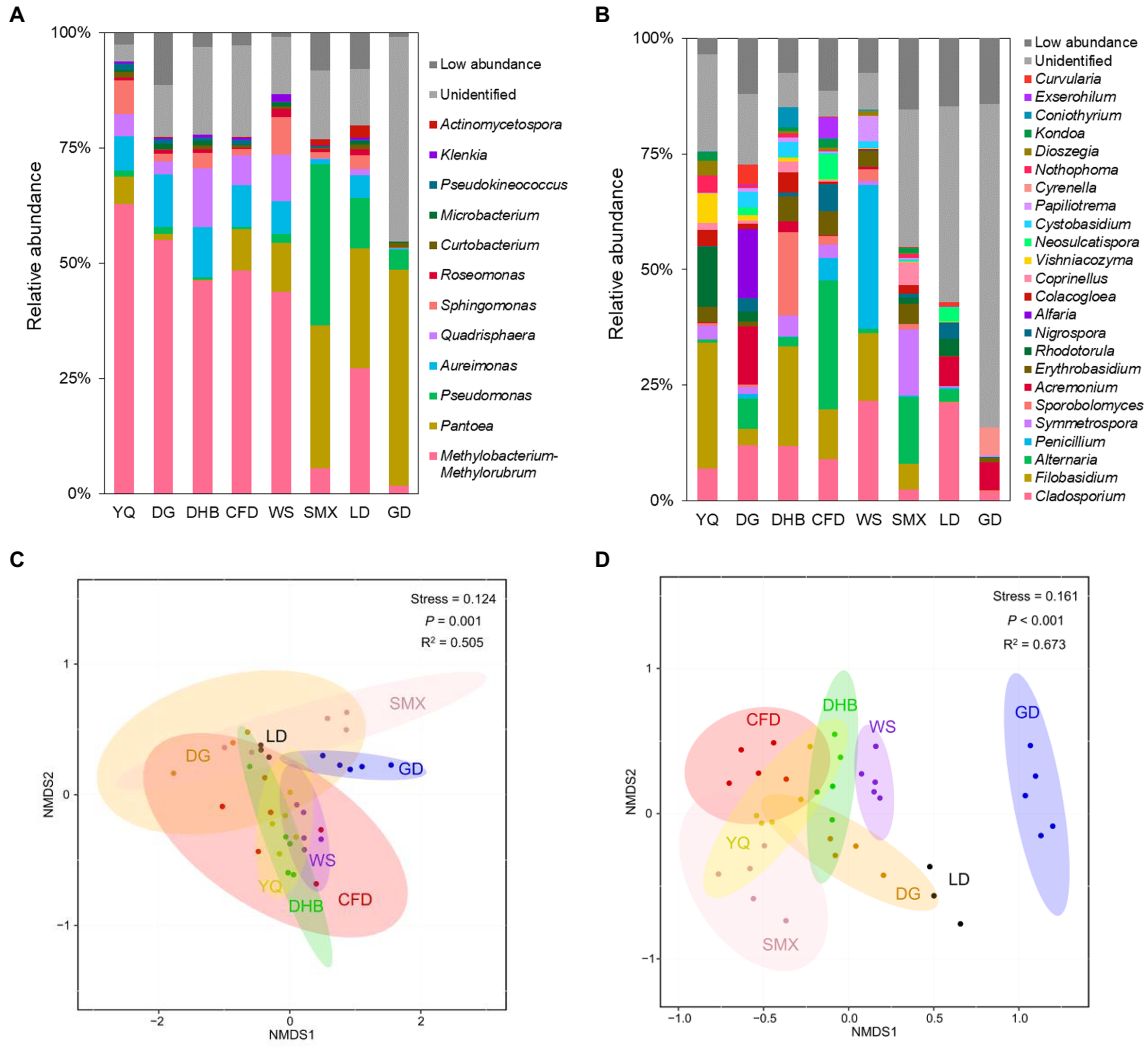
Relative abundance at genus level of bacterial taxa **(A)** and fungal taxa **(B)** in the foliar microbiome of eight genotypes of wild soybean. The total relative abundance of low-abundant bacteria and fungi that accounted for less than 0.5% is indicated as low abundance in darkgrey, and unidentified taxonomic groups are indicated in light grey. At each sampling site, there were three to five technical replicates sampled from three to five plants with the same genotype. Abbreviations for the sample name are depicted in Table S1. The NMDS analysis of bacterial **(C)** and fungal **(D)** community at genus level based on Bray-Curtis distances categorized by host genotypes.

At the phylum level, the foliar bacterial community of wild soybean was dominated by Proteobacteria (80.97 to 98.10%) and Actinobacteriota (1.39 to 18.62%), and the foliar fungal community was dominated by Ascomycota (27.85 to 93.67%) and Basidiomycota (6.02 to 71.97%) (Supplementary Figure S2). At the genus level, the foliar bacterial community was dominated by *Methylobacterium-Methylorubrum* (1.72 to 62.78%), *Pantoea* (0.10 to 46.92%), *Pseudomonas* (0.54 to 34.98%), *Aureimonas* (0.36 to 11.46%), and *Quadrisphaera* (0.05 to 12.82%) (Figure 1A), while the foliar fungal community was dominated by *Cladosporium* (1.74 to 21.58%), *Filobasidium* (0.001 to 27.16%), *Alternaria* (0.001 to 27.91%), *Penicillium* (0.02 to 31.14%), and *Symmetrospora* (0.05 to 14.33%) (Figure 1B). However, there were significant fluctuations in the microbial community composition among various genotypes and sampling sites. For the α diversity, the bacterial Shannon index (Kruskal-Wallis test, *p* = 0.023) and richness (Kruskal-Wallis test, *p* = 0.027) differed significantly among host genotypes (Supplementary Figures S3A,C); in addition, a significant difference was observed for fungal richness among host genotypes (Scheffe’s test, *p* = 0.001) (Supplementary Figure S3D). When comparing the samples between the northern and southern regions, the richness of fungal communities was significantly different, while no discernible variation was observed in bacterial communities (Supplementary Table S6). The non-metric multidimensional scaling (NMDS) ordinations revealed a clear separation among host genotypes for both bacteria and fungi (bacteria: *R*^2^ = 0.505, *p* = 0.001; fungi: *R*^2^ = 0.673, *p*<0.001) (Figures 1C,D). This separation was also observed for microbial communities between the northern and southern regions (bacteria: *R*^2^ = 0.486, *p* = 0.001; fungi: *R*^2^ = 0.632, *p* = 0.001) (Supplementary Figure S4).

### 3.3. Host genotype and environment factors explained the foliar bacterial and fungal communities

**Figure 2:**
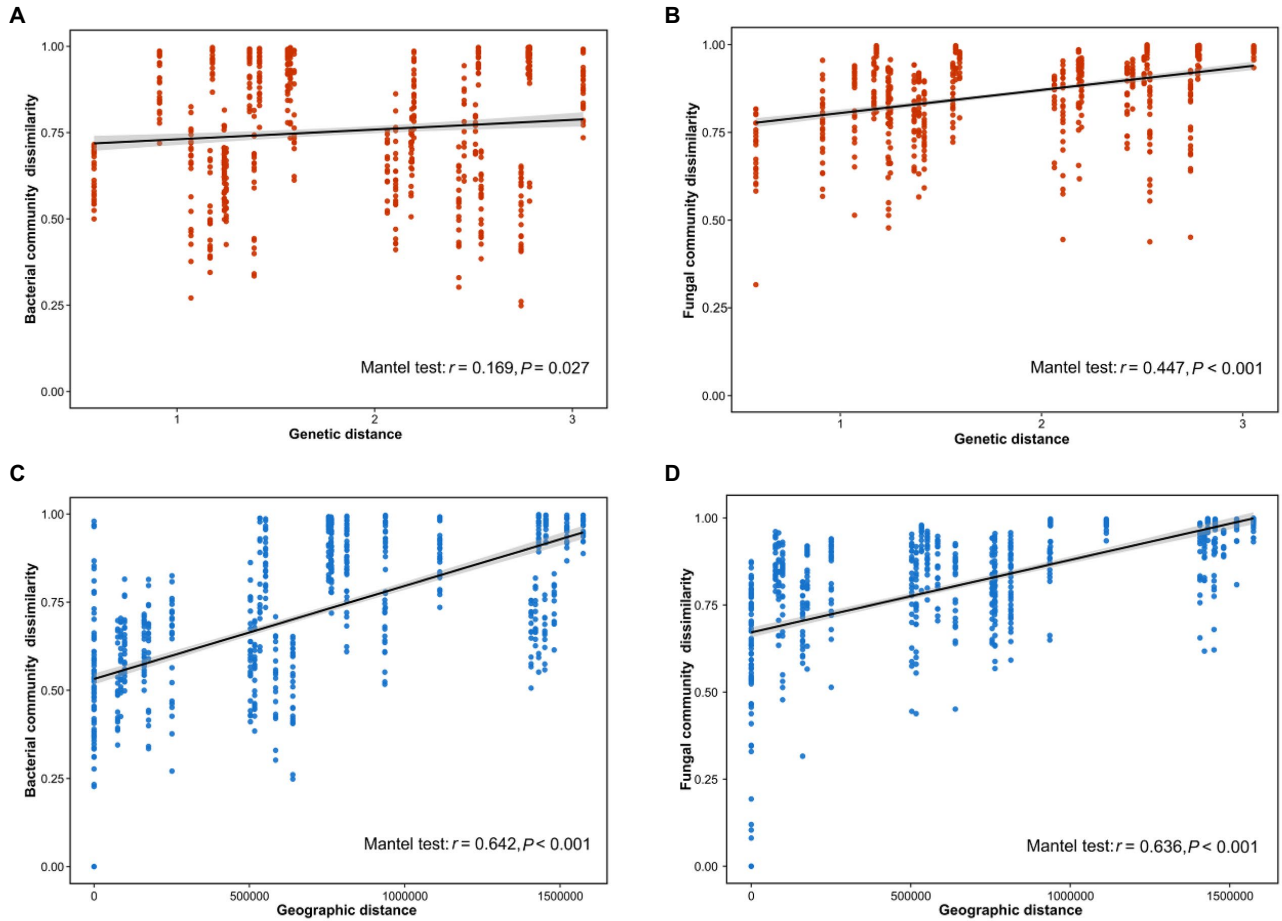
Correlation between the dissimilarity of the bacterial **(A)** and fungal **(B)** communities and host genetic distance. The distance-decay relationships between dissimilarity of the bacterial **(C)** and fungal **(D)** communities and geographical distance.

**Figure 3:**
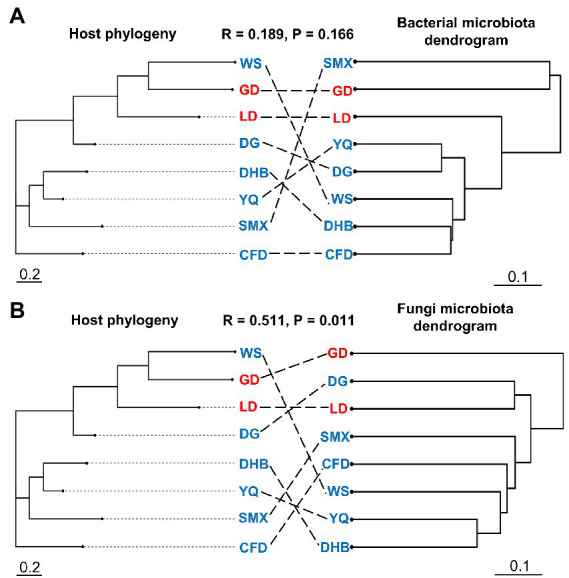
The neighbor-joining tree of wild soybean populations (left panel) and dendrogram of hierarchical clustering of Bray–Curtis dissimilarity of the bacterial **(A)** and fungal **(B)** communities (right panel). The eight genotypes of the microbial community were averaged using biological replicates.

Our results indicated that the host genotype could contribute to the explanation of the foliar microbiome variation. The genetic distance was significantly correlated with bacterial (*r* = 0.169, p = 0.027) and fungal dissimilarity (*r* = 0.447, *p* < 0.001) (Figures 2A,B). Furthermore, the geographic distance was significantly positively correlated with discrepancies in both bacterial (*r* = 0.642, *p* < 0.001) and fungal (*r* = 0.636, *p* < 0.001) communities (Figures 2C,D). Additionally, we further analyzed the correlations between host genotypic phylogeny and foliar microbiome phylogeny. The results revealed that there was a significantly positive correlation between the phylogenetic relationship of wild soybean and the fungal dissimilarity (*r* = 0.511, *p* = 0.011), while no significant correlation was observed between bacterial dissimilarity and the host phylogeny (*r* = 0.189, *p* = 0.166) (Figures 3A,B).

**Figure 4:**
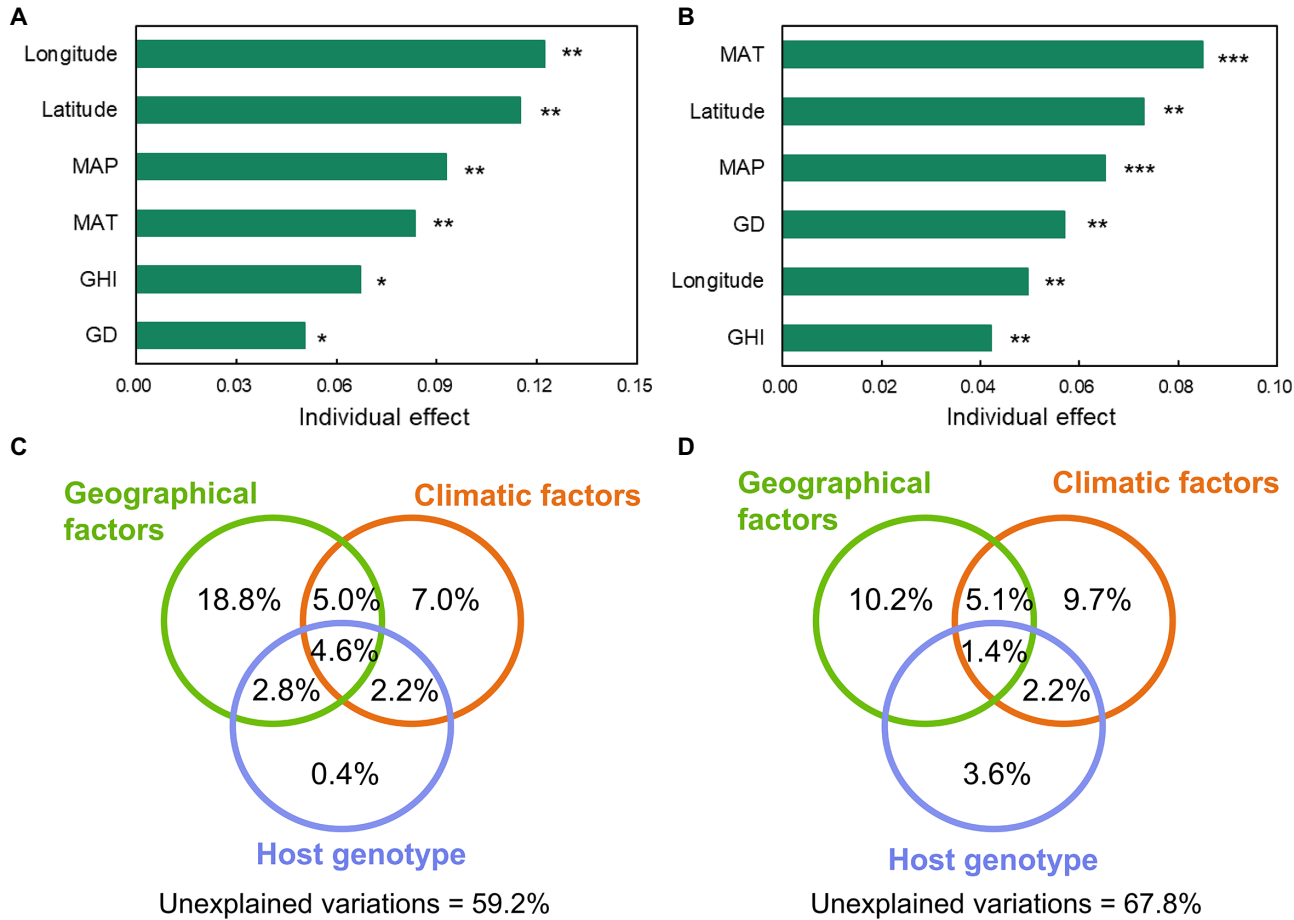
The individual effect of the environmental and host genetic distance variables to explain the variation of bacterial **(A)** and fungal **(B)** communities at genus level in the foliar microbiome of wild soybean (“*,” 0.01 < *p* < 0.05, “**,” 0.01 < *p* < 0.001, “***,” *p* < 0.001). The relative importance of individual variables was calculated using *rdacca.hp* package. GD, genetic distance; MAP, mean annual precipitation; MAT, mean annual temperature; and GHI, global horizontal irradiance. The effects of explanatory variables (geographical, climatic, and host genotype) on bacterial **(C)** and fungal **(D)** communities were estimated using variation partition analysis. Shared effects are indicated by the overlap of circles. Geographical factors include longitude and latitude. Climatic factors include MAP, MAT, and GHI. The host genotype represents the host genetic distance.

According to RDA at the genus level, all explanatory variables, including geographical factors (latitude and longitude), climatic factors (MAT, MAP, and GHI), and host genotype (host genetic distance), were significantly correlated with the assembly of the foliar microbial community (Supplementary Figures S5A,B). Hierarchical partitioning was performed based on RDA to further investigate the contributions of these factors to the variation of the foliar microbial community. The results revealed that longitude, latitude, mean annual precipitation (MAP), mean annual temperature (MAT), global horizontal irradiance (GHI), and genetic distance explained 12.2, 11.5, 9.3, 8.4, 6.7, and 5.0% of the variation of bacterial communities, respectively; similar variables explained the variation of fungal communities by 5.0, 7.3, 6.5, 8.5, 4.2, and 5.7% (Figures 4A,B). The aforementioned explanatory variables were thereafter classified into geographical, climatic, and host genotype categories using variation partition analysis (Figures 4C,D). Results demonstrated that the pure environmental parameters (including geographical and climatic factors) and host genotype explained 25.8 and 0.4% for bacterial communities and 19.9 and 3.6%, respectively, for fungal communities. There were shared effects between the associated variables. For instance, the shared effects between geographic location and host genotype explained 2.8% of the variation in bacteria, while the shared effects between climatic factors and host genotype explained 2.2% of the variation in bacteria and fungi. These results indicated that the distribution of plant genotypes was influenced by both geographic and climatic factors in an interacting manner. For the bacterial and fungal communities, the unexplained variations were 59.2 and 67.8%, respectively. These results indicated that the environmental factors and host genotype played a significant role in determining the foliar microbiome.

### 3.4. The core foliar microbiome of the wild soybean and its driving factors

**Figure 5:**
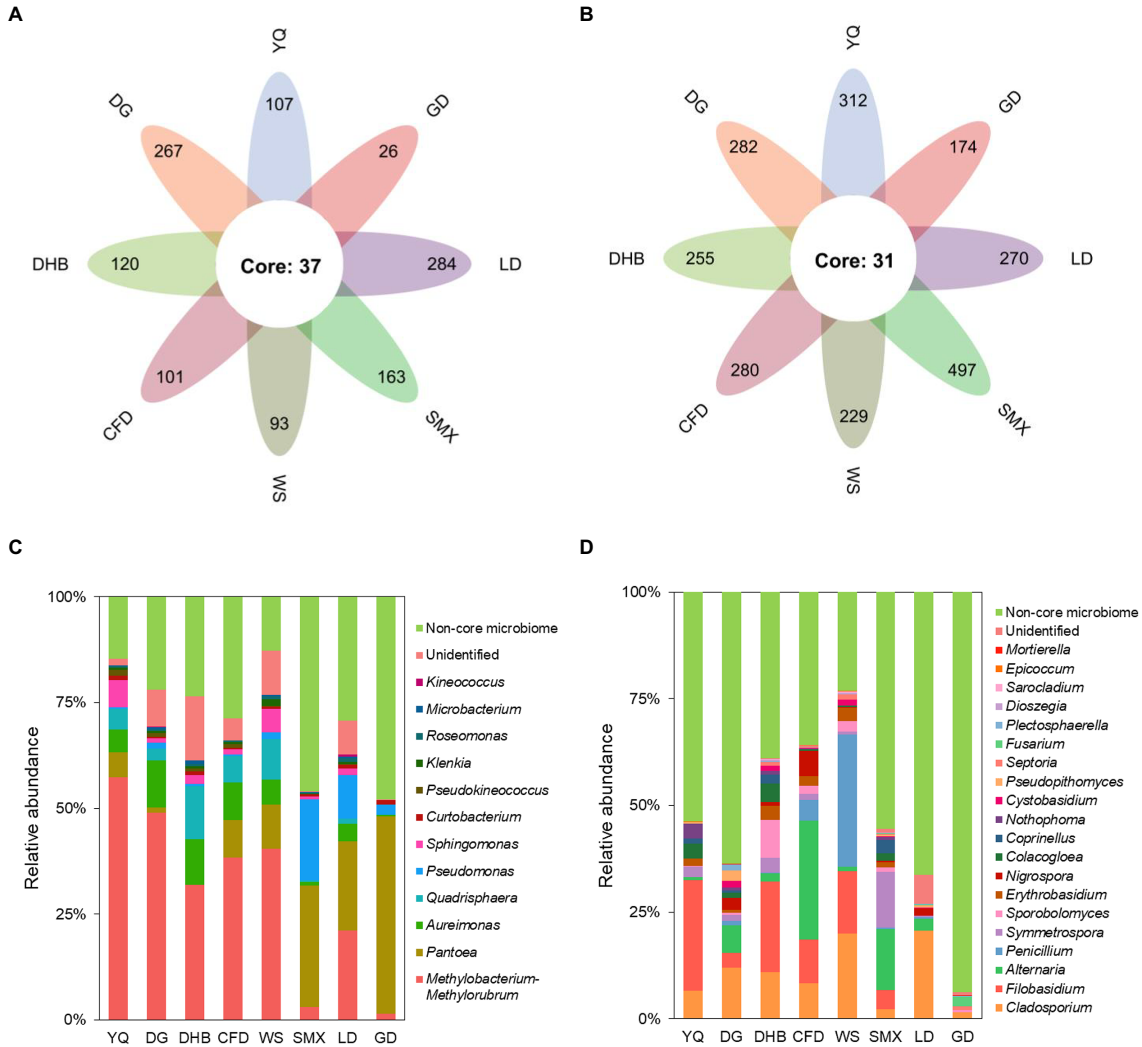
The core microbes of eight genotypes of bacterial **(A)** and fungal **(B)** communities. The relative abundance at the genus level of the core bacterial **(C)** and fungal **(D)** taxa in the foliar microbiome of wild soybean. The non-core taxa are indicated in green.

By the establishment of the prevalence threshold of 70%, the core bacterial and fungal ASVs of wild soybean were identified. Among the total 2,058 bacterial and 4,489 fungal ASVs obtained, we found 37 bacterial and 31 fungal ASVs to be consistently prevalent across all of the eight genotypes (Figures 5A,B). Even though this core microbiome only accounted for 1.80% of all bacterial and 0.69% of all fungal ASVs, its relative abundance (RA) was high, accounting for 71.91 and 46.17%, respectively (Figures 5C,D). The core bacterial microbiome was dominated by Proteobacteria, which represented 65.04% of the overall bacterial RA on average. Within Proteobacteria, the genus *Methylobacterium-Methylorubrum* (with nine ASVs, and an RA of 30.33%), *Pantoea* (with three ASVs, and an RA of 15.40%), *Aureimonas* (with six ASVs, and an RA of 5.91%), *Pseudomonas* (with one ASV, and an RA of 4.54%), and *Sphingomonas* (with six ASVs, and an RA of 2.31%) dominated the bacterial community (Figure 5C). According to the fungal core microbiome, *Ascomycota* was the most dominant phylum, and its RA accounted for 26.80% of the total fungi. Within *Ascomycota*, *Cladosporium* (with three ASVs, and an RA of 10.25%), *Alternaria* (with one ASV and an RA of 6.88%), *Penicillium* (with one ASV and an RA of 4.72%), and *Nigrospora* (with three ASVs, and an RA of 1.47%) were the dominant genera (Figure 5D).

**Figure 6:**
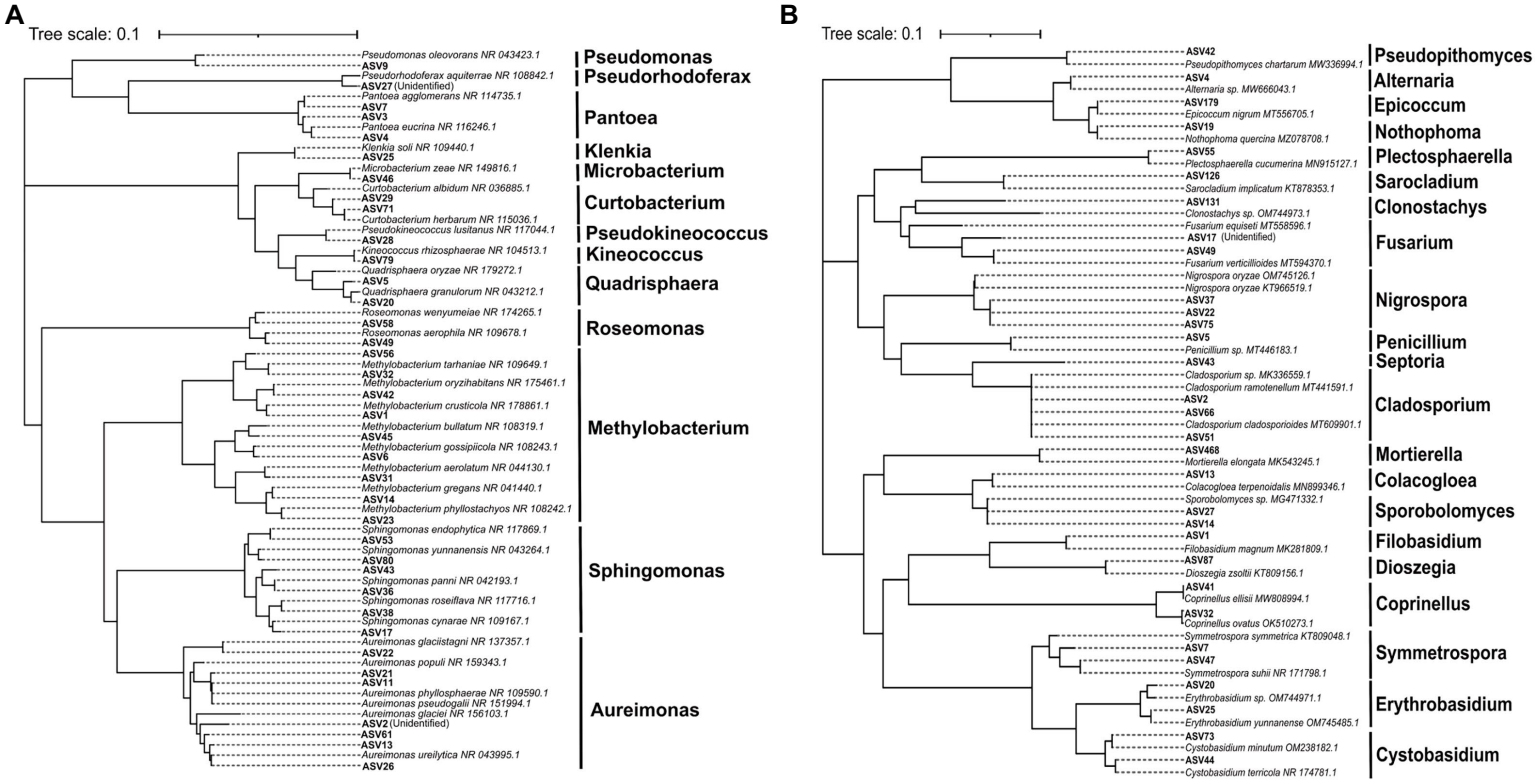
The neighbor-joining (NJ) phylogenetic tree showing the core bacterial **(A)** and fungal **(B)** ASVs of the foliar microbiome of wild soybeans.

The phylogenetic tree of the core foliar microbes and the microbes that possess high similarity to the core foliar microbes was constructed at the genus level to further reveal the taxonomic information of the core microbiome (Figures 6A,B). Results revealed that the core bacteria included *Aureimonas*, *Sphingomonas*, and *Methylobacterium*, and the core fungi included *Nigrospora*, *Penicillium*, *Septoria*, and *Cladosporium*. Although the relative abundance of the core taxa was different across genotypes and geographical conditions (Supplementary Figures S6A–C), these taxa might have coevolved with the wild progenitor of soybean, indicating that their potential function is important for the wild soybean health. Results of the RDA revealed that latitude, which accounted for 16.3% of the core bacterial community assembly, and MAT, which accounted for 8.3% of the core fungal community assembly, were the two most significant factors. However, several other variables, including longitude, MAP, GHI, and host genetic distance, also significantly influenced the core foliar microbiome (Supplementary Figures S7A–D). Similarly, the environmental factors strongly influenced the core foliar bacterial and fungal communities in comparison to the host genotype (Supplementary Figures S7E,F).

## 4. Discussion

### 4.1. Host genotype and environmental conditions influence the foliar microbiome of wild soybeans

According to our research, both genetic distance and geographic conditions were significantly correlated with the foliar microbial community of wild soybeans. These findings indicate that host genotype and environmental conditions might play a significant role in driving the foliar microbial community assembly in the phyllosphere of *G. soja*, the wild progenitor of soybean, which is economically and culturally an important crop in China.

We found that *G. soja* sampled from eight sites across China has distinct genotypes, and the *G. soja* genotypes differ essentially along with the latitude of sampling sites (Supplementary Figure 1B). However, there were notable exceptions too, such as the genotypes from southern LD and GD were phylogenetically clustered with the northern group. This result might be related to the origin and dispersal of wild soybean. It could be possible that wild soybeans growing in the south of the Yangtze River might have spread from the Northeast region and Yellow River Valley (Dong et al., 2001). Further, the geographic exceptions might have been caused by the long-distance migration of human transportation and also the transportation of birds and other animals (Nathan et al., 2008).

Genotypes of wild soybean exhibited a significant impact on the community composition and structure of the foliar microbiome, even after accounting for climate and substantial patterns in the spatial distribution (Figures 1A–D). This result is in accordance with previous findings revealing that host genotype affects the rhizosphere community in soybean (Zhong et al., 2019). The dissimilarity of the foliar microbial community was significantly associated with the host genetic distance (Figures 2A,B). This phenomenon was consistent with several recent investigations (Cordier et al., 2012; Qian et al., 2018). Further, substantial genetic differentiation and limited gene flow were detected among wild soybean natural populations (He et al., 2012), and our study indicated that the host genetic distance could help in explaining the foliar community assembly of *G. soja*. It is generally recognized that plants with different genotypes could develop different phenotypes, and these phenotypes could shape their phyllosphere microbiota (Li et al., 2018). For instance, leaf structure (Bodenhausen et al., 2014; Aragon et al., 2017), leaf exudates and volatiles (Kolton et al., 2013; Farre-Armengol et al., 2016), and plant defense signaling pathways (Kniskern et al., 2007) are all key role in shaping phyllosphere communities. In addition, a recent study reported that the phyllosphere microbial community is also influenced by vertical dispersal, and a part of microbes from the embryo could be transmitted to the phyllosphere from the seed to the seedling (Abdelfattah et al., 2021b). In our study, the host genotype is an influential driving factor in the foliar microbial community variation of wild soybeans.

The bacterial and fungal community composition and richness also differed among the eight sampling sites (Figures 1A,B; Supplementary Figures S3C,D) sampled across climatic gradients. According to the distance-decay results, the foliar bacterial and fungal community dissimilarities were positively correlated with geographic distance among sampling sites (Figures 2C,D). All of these findings indicated that the local environmental conditions could be another significant factor in shaping the foliar microbial community of wild soybeans. Our results are consistent with earlier studies (Vogel et al., 2021). In general, the microbial communities colonizing the same host species and/or organs could be filtered and selected by environmental conditions, such as temperature, heat, and precipitation, which could directly shape the foliar microbial community (Jiao et al., 2019; Chen et al., 2021). For example, under drought stress, the diversity of the phyllosphere bacterial community of grass diminished, and Gammaproteobacteria replaced other taxa as the dominant species (Bechtold et al., 2021). In addition, local dispersal from neighboring plants could also be an important factor influencing foliar microbial community assembly (Meyer et al., 2022). This influence contributes to the geographical distance-decay relationships (Figures 2C,D). Furthermore, geographical and climatic factors could indirectly influence the foliar microbial community by influencing the distribution of host genotypes, as discussed above. Therefore, our results demonstrated that environmental conditions could also affect the foliar microbial composition of wild soybeans.

### 4.2. The core foliar microbiota is potentially beneficial to wild soybean growth

Although the foliar microbial communities are significantly different depending on environmental conditions and host genotypes, the core microbes with high abundances were present across all the samples. Increased abundance and occurrence of the core foliar microbiota suggest their significance to the plants. Some core foliar microbiota might assist the hosts in acquiring nutrients. For instance, the core microbes of *Methylobacterium*, *Pantoea*, *Pseudomonas*, and *Sphingomonas*, with the relative abundance of 30.33, 15.4, 4.54, and 2.31%, respectively, were reported as N_2_-fixing bacteria (Loiret et al., 2004; Albino et al., 2006), and some strains of *Pseudomonas* have also been observed to assist wheat in absorbing phosphorus (Zabihi et al., 2011). Additionally, there are also some microbial taxa that could help the host plants in increasing their resistance against abiotic stresses. Some species of *Pseudomonas* detected in plant phyllosphere have been demonstrated to be resistant to mercury contamination (Durand et al., 2018). The bacterial genera of *Curtobacterium* and *Microbacterium,* as well as the fungal genera of *Cladosporium* and *Alternaria* that colonized the leaf surface of *Euonymus japonicus* were all tolerant of ozone stress (Liu et al., 2022), and all of these genera were core foliar microbiota of wild soybean (Figures 5C,D). Moreover, some core microbes of wild soybean might involve in assisting the host to resist the biotic stresses. The core microbes of *Methylobacterium* spp. strains (an RA of 30.33%) and *Sphingomonas* spp. strains (an RA of 2.31%) (Figure 5C) have been reported to protect hosts against pathogens (Innerebner et al., 2011; Ardanov et al., 2012). Furthermore, certain microbial species have been cultivated as bio-control agents for reducing crop diseases or restraining weeds. For example, some members of *Penicillium* could effectively prevent verticillium wilt by inducing resistance in tomatoes (Larena et al., 2003), and *Alternaria* is capable of controlling weeds to protect the health of agro-ecosystems (Charudattan, 2001).

In addition to the aforementioned beneficial microbial taxa, some taxa that could cause plant disease were also found in this study. For instance, certain members of bacterial genera (e.g., *Curtobacterium* and *Pseudomonas*) as well as fungal genera (e.g., *Alternaria* and *Penicillium*) were identified as potential pathogens for pear and apple bark (Arrigoni et al., 2018). Meanwhile, *Nigrospora oryzae* and *Plectosphaerella cucumerina* were also the core fungal microbiota of *G. soja*, which have the potential to cause leaf spot and root rot, respectively (Chen et al., 2019; Elmer et al., 2020). Nevertheless, it should be mentioned that although the sequences of the core microbial ASVs were identified by clustering with the known sequences from NCBI GenBank in accordance with their phylogenetic relationships (Figures 6A,B), it is difficult to differentiate the taxa below genus level using 16S-based profiling. Therefore, to increase the beneficial microorganisms and restrain the detrimental ones for soybean production, more precise sequencing tools should be employed in the future to analyze the functions and roles of the foliar microbiome of wild soybean.

## 5. Conclusion

We investigated the foliar microbial community structures of wild soybean in various genotypes across large-scale regions of China and the driving factors of microbial community assembly. The results revealed that both environmental conditions, as well as host genotypes, were crucial drivers in shaping the foliar microbiome of wild soybean, and the host genetic distance played a significant role in explaining the assembly of the foliar microbiome. The identified core foliar microbiota was present in various wild soybeans with an increased abundance, and they were potentially beneficial to the health of the host; they were also significantly influenced by the aforementioned two factors. This study expanded the knowledge of driving factors in foliar microbiome of wild soybeans, and the results would be helpful for developing strategies for the agricultural management of cultivated soybeans. Together, our results suggested that we could indirectly affect the microbiome of soy across broad climatic regions with implications for our ability to deal with the impacts of ongoing climatic changes on crop microbiomes.

## Data availability statement

The datasets presented in this study can be found in online repositories. The names of the repository/repositories and accession number(s) can be found at: https://www.ncbi.nlm.nih.gov/genbank/, PRJNA862265.

## Author contributions

Y-GZ, MD-B, G-LD, and PG-P contributed to conception and design. RZ, GY, H-LC, YY, MY, X-YY, and LL contributed to acquisition of samples, data, analysis and interpretation of data. RZ, GY, G-LD, MD-B, and Y-GZ contributed to drafting the article or revising the manuscript. All authors contributed critically to the drafts and gave final approval for publication.

## Funding

This study was supported by the National Natural Science Foundation of China (grant no. 41991332 and 42090063). MD-B acknowledges support from the Spanish Ministry of Science and Innovation for the I+D+i project PID2020-115813RA-I00 funded by MCIN/AEI/10.13039/501100011033. MD-B is also supported by a project of the Fondo Europeo de Desarrollo Regional (FEDER) and the Consejería de Transformación Económica, Industria, Conocimiento y Universidades of the Junta de Andalucía (FEDER Andalucía 2014–2020 Objetivo temático “01 – Refuerzo de la investigación, el desarrollo tecnológico y la innovación”) associated with the research project P20_00879 (ANDABIOMA).

## Conflict of interest

The authors declare that the research was conducted in the absence of any commercial or financial relationships that could be construed as a potential conflict of interest.

## Publisher’s note

All claims expressed in this article are solely those of the authors and do not necessarily represent those of their affiliated organizations, or those of the publisher, the editors and the reviewers. Any product that may be evaluated in this article, or claim that may be made by its manufacturer, is not guaranteed or endorsed by the publisher.

## Footnotes

^1^ https://www.resdc.cn/

^2^ https://www.arb-silva.de/

^3^ https://blast.ncbi.nlm.nih.gov

^4^ https://itol.embl.de/
