# Supplementary material for: Environmental factors and host genotype control foliar epiphytic microbial community of wild soybeans across China
Source: Front Microbiol. 2023 Mar 13;14:1065302. doi: 10.3389/fmicb.2023.1065302 (PMC10041966; doi:10.3389/fmicb.2023.1065302)
Supplement: Supplementary file 1 [file Data_Sheet_1.doc]

***Supplementary Material***

**Environmental factors and host genotype control foliarepiphytic microbial community of wild soybeans across China**

Rui Zhou ^1,2^, Gui-Lan Duan ^1,2^, Pablo García-Palacios ^3^, Guang Yang ^1^, Hui-Ling Cui ^1,2^, Ming Yan ^1,2^, Yue Yin ^1,2^, Xing-Yun Yi ^1,2^, Lv Li ^1,2^, Manuel Delgado-Baquerizo ^4,5^ and Yong-Guan Zhu ^1,2,6*^

^1^ State Key Laboratory of Urban and Regional Ecology, Research Center for Eco-Environmental Sciences, Chinese Academy of Sciences, Beijing, China

^2^ University of Chinese Academy of Sciences, Beijing, China

^3^ Instituto de Ciencias Agrarias, Consejo Superior de Investigaciones Científicas, Madrid, Spain

^4^ Laboratorio de Biodiversidad y Funcionamiento Ecosistémico, Instituto de Recursos Naturales y Agrobiología de Sevilla (IRNAS), CSIC, Sevilla, Spain

^5^ Unidad Asociada CSIC-UPO (BioFun), Universidad Pablo de Olavide, Sevilla, Spain

^6^ Institute of Urban Environment, Chinese Academy of Sciences, Xiamen, China

**Additional file contains:**

Figure S1-S7

Table S1-S6


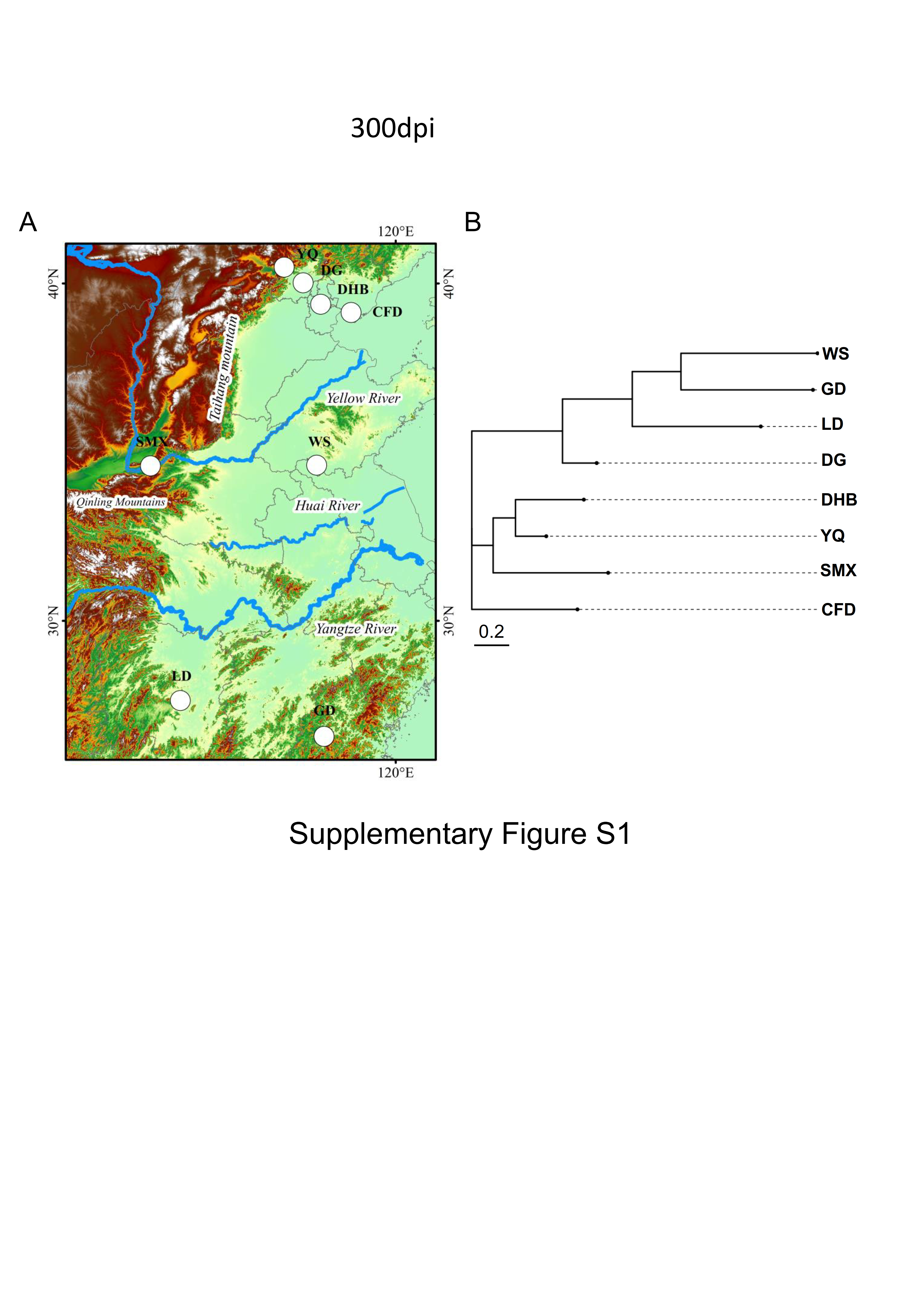


**Figure S1**. Sampling distribution of *G. soja* across northern and southern regions of China **(A)**. Sampling sites are represented by white points. A neighbor-joining tree of host populations based on Nei’s genetic distance **(B)**.


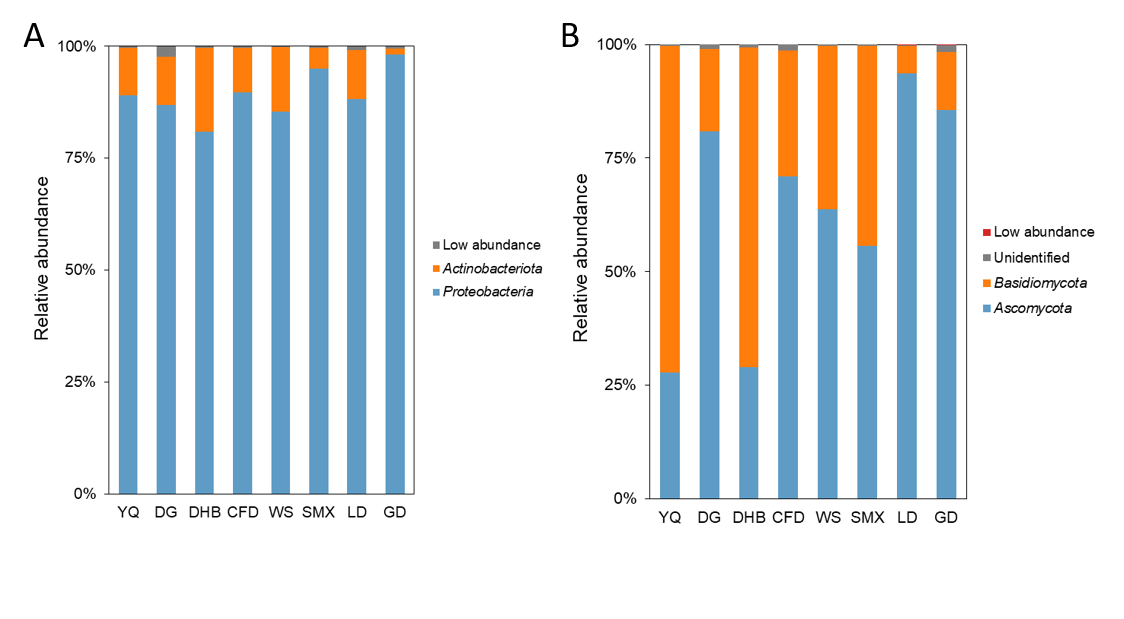


**Figure S2.** The relative abundance at the phylum level of bacterial **(A)** and fungal **(B)** taxa in the foliar microbiome of eight genotypic wild soybeans. The total relative abundance of low-abundant bacteria and fungi that accounted for less than 0.5% is indicated in dark grey.





**Figure S3.** The bacterial **(A)** and fungal **(B)** alpha-diversity in each genotype was assessed by Shannon. The bacterial **(C)** and fungal **(D)** community richness of different genotypes. The richness of microbial communities refers to the quantity of observed species.


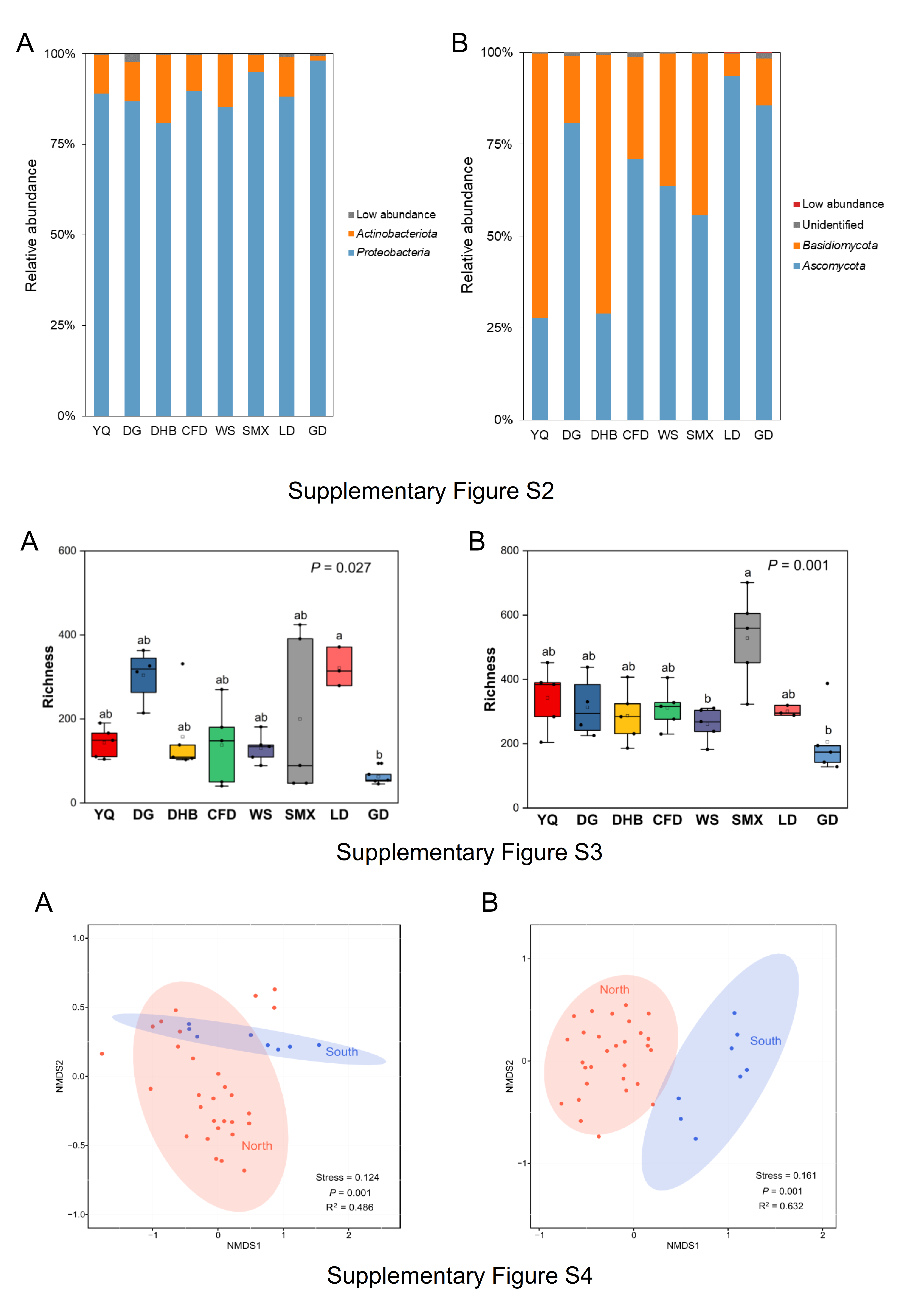


**Figure S4.** The NMDS analysis of the bacterial **(A)** and fungal **(B)** communities at the genus level based on Bray-Curtis distances categorized by the northern and southern regions





**Figure S5.** RDA indicates the genus-level driving factors for bacterial **(A)** and fungal **(B)** assembly. The samples (37) are represented by black dots. GD, genetic distance; MAP, mean annual precipitation; MAT, mean annual temperature; and GHI, global horizontal irradiance.


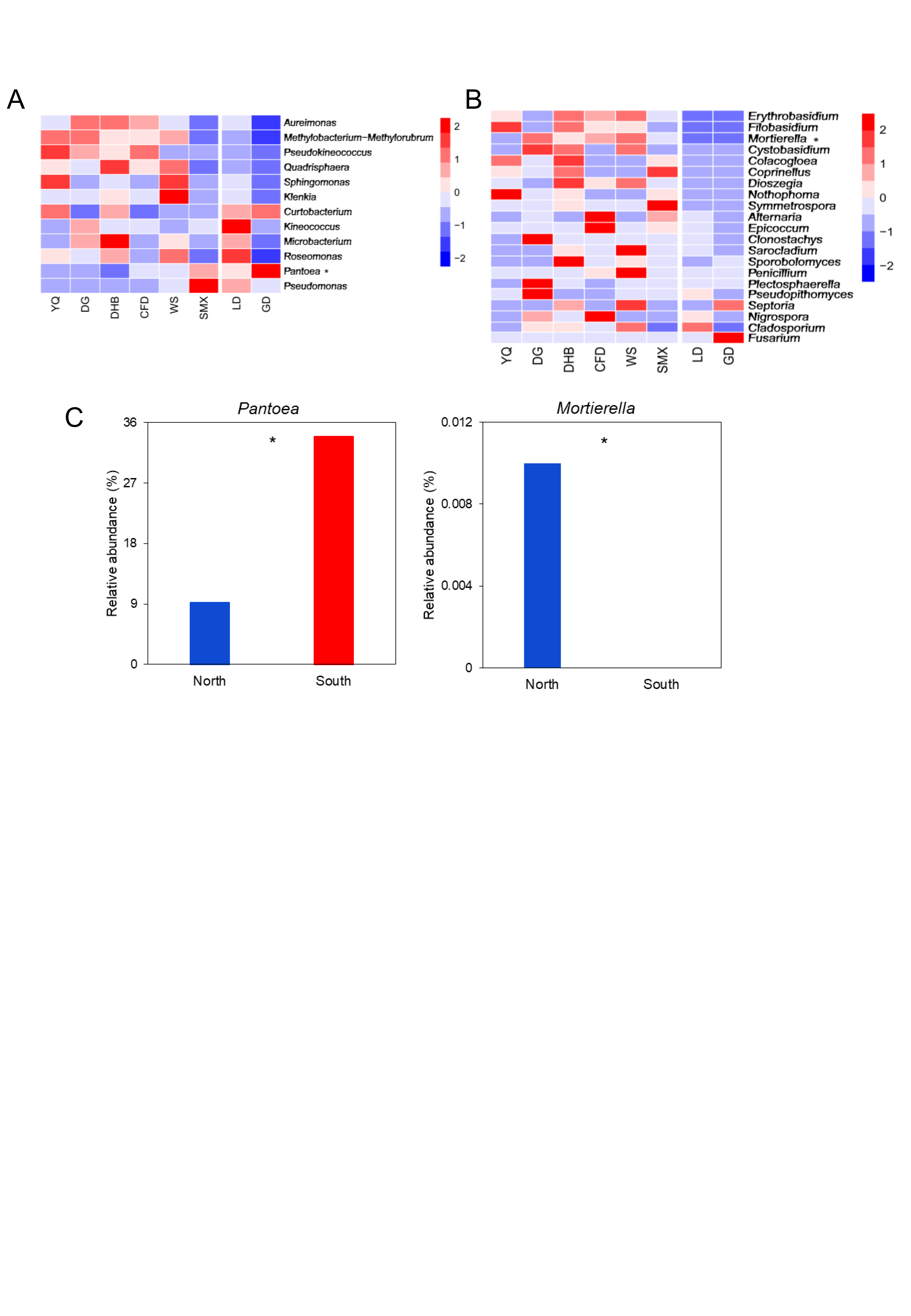


**Figure S6.** Heatmap analysis of core bacterial **(A)** and fungal **(B)** taxa at genus level across genotypes. The closer the color to red, the higher the relative abundance of the core microbial taxa in the corresponding sample. The closer the color to blue, the lower the relative abundance of these taxa in the samples. The eight genotypes of the microbial community were averaged by biological replicates. The bacterial and fungal taxa with significantly different relative abundance between northern and southern regions **(C)** (“*”, p < 0.05, Scheffe’s test, and Mann-Whitney *U* test).





**Figure S7**. RDA illustrates geographic location, climate factors, and host genotypes that influence core bacterial **(A)** and fungal **(B)** assembly at the genus level. The samples (37) are represented by black dots. The individual effect of the environmental and host genetic distance variables to explain the core bacterial **(C)** and fungal **(D)** community variation at genus level in the foliar microbiome of wild soybean (“*”, 0.01 < p < 0.05, “**”, 0.01 < p < 0.001, “***”, p < 0.001). GD, genetic distance; MAP, mean annual precipitation; MAT, mean annual temperature; and GHI, global horizontal irradiance. The influences of explanatory variables (geographical, climatic, and host genetic factors) on bacterial **(E)** and fungal **(F)** communities were estimated using variation partition analysis. Shared effects are indicated by the overlap of circles. Geographical factors include longitude and latitude. Climatic factors include MAP, MAT, and GHI. The host genotype represents the genetic distance of the host.

**Table S1** Details of the sampling sites of *Glycine soja*.

| Populations | Localities | Latitude (N) | Longitude (E) | Number of samples/ technical replicate |
| --- | --- | --- | --- | --- |
| YQ | Yanqing, Beijing | 40.45 | 115.95 | 5 |
| DG | Shunyi, Beijing | 40.02 | 116.64 | 4 |
| DHB | Wuqing, Tianjing | 39.42 | 117.28 | 5 |
| CFD | Tangshan, Hebei | 39.19 | 118.38 | 4 |
| WS | Zaozhuang, Shandong | 34.77 | 117.14 | 5 |
| SMX | Sanmenxia, Henan | 34.75 | 111.11 | 5 |
| LD | Loudi, Hunan | 27.47 | 112.19 | 3 |
| GD | Sanming, Fujian | 26.31 | 117.40 | 4 |

Note: The leaves collected from a single plant were recorded as a technical replicate.

**Table S2** Climatic information of sampling sites.

| Sites | MAT | MAP | GHI |
| --- | --- | --- | --- |
| YQ | 9.1 | 423.7 | 1489.5 |
| DG | 11.6 | 610.1 | 1411.0 |
| DHB | 11.9 | 590.6 | 1416.4 |
| CFD | 11 | 616.5 | 1459.9 |
| WS | 13.1 | 666.3 | 1389.1 |
| SMX | 13.7 | 662.6 | 1380.3 |
| LD | 16.3 | 1342.1 | 1191.4 |
| GD | 18.7 | 1657.7 | 1361.3 |

Note: Mean annual precipitation (MAP), mean annual temperature (MAT) and global horizontal irradiance (GHI).

**Table S3** Primer sequences of microsatellite molecular markers (Simple Sequence Repeats, SSR) for wild soybean populations.

| Primer | Linkage group | Primer sequence 5’—3’ |
| --- | --- | --- |
| satt468 | D1a (Gm1) | Forward: GCGTCTCTTATTTTGACCTTTTTAACTT |
|  |  | Reverse: GCGTTTTGTATTTGGTCTATCTGCTTAG |
| satt005 | D1b (Gm2) | Forward: TATCCTAGAGAAGAACTAAAAAA |
|  |  | Reverse: GTCGATTAGGCTTGAAATA |
| satt675 | N (Gm3) | Forward: GCGCTATTTCCGTCCTATTATCATTTTCGTC |
|  |  | Reverse: GCGTCTAACACGTATTTATTATTGGTCAATT |
| satt338 | C1 (Gm4) | Forward: GCGCCCAAGTATTATGAGATATTTGAT |
|  |  | Reverse: GCGATAATTTTAAAACTGGACCA |
| satt591 | A1 (Gm5) | Forward: GCGCGACCTTAATGATA |
|  |  | Reverse: GCGCCCAAAGCTTAAAATTTAATA |
| satt202 | C2 (Gm6) | Forward: GGAATGCATGAGTATTAACCTCTTAT |
|  |  | Reverse: GGGCTAACGAACATGTAACTTATCAAC |
| satt201 | M (Gm7) | Forward: GCGTTGATACTTTCCTAAGACAAT |
|  |  | Reverse: GGGAGAGAAGGCAATCTAA |
| satt421 | A2 (Gm8) | Forward: CAAAGCTTCTTCTTCTTCT |
|  |  | Reverse: CACAGGTTGAGACAAAACAAA |
| satt326 | K (Gm9) | Forward: AGATTCTCCTTTGCTTCTTAGT |
|  |  | Reverse: GTTAGTTCACCTTCCAGTATTTGA |
| satt153 | O (Gm10) | Forward: GGGTTATATCAGTTTTTCTTTTTGTT |
|  |  | Reverse: CCATCCTCGTTAGCATCTAT |
| satt251 | B1 (Gm11) | Forward: CCTCCACCCCCTTCCCACCCAAAA |
|  |  | Reverse: GGTGATATCGCGCTAAAATTA |
| satt052 | H (Gm12) | Forward: GAATAAAATTAGGATAAGTGATAAG |
|  |  | Reverse: ACAGAAAAAAGAAAATGTCA |
| satt144 | F (Gm13) | Forward: CGTCGCCATCACTATGAGAA |
|  |  | Reverse: CCATCTTGAGCAGAGTTTGAAGTT |
| satt304 | B2 (Gm14) | Forward: GGGTAGTGACGTATTTCATGGTC |
|  |  | Reverse: GCGTAAAAACATTCGTTGACTACATAA |
| satt185 | E (Gm15) | Forward: GCGCATATGAATAGGTAAGTTGCACTAA |
|  |  | Reverse: GCGTTTTCCTACAATAATATTTCAT |
| satt380 | J (Gm16) | Forward: GCGAGTAACGGTCTTCTAACAAGGAAAG |
|  |  | Reverse: GCGTGCCCTTACTCTCAAAAAAAAA |
| satt461 | D2 (Gm17) | Forward: AAATACAAGCTTTAATAAAGTGCAGA |
|  |  | Reverse: CTTACGTTTCCATAGATTTCTCG |
| satt504 | G (Gm18) | Forward: GCGCATGTGCAACTTGAAAAACA |
|  |  | Reverse: TCGTTGGTTGACCCAATGTCATC |
| satt481 | L (Gm19) | Forward: GGGTTAACCGTCCACACATCTATT |
|  |  | Reverse: GACGGTTTTAAACGGTAAGAAAAT |
| sat_421 | I (Gm20) | Forward: GCGTGAAGCCGCACCAATA |
|  |  | Reverse: GCGAACTCCTACTATAATG |

**Table S4** Genetic distance among eight populations of *G. soja.*

| Population | GQ | WS | DHB | YQ | SMX | CFD | GD | LD |
| --- | --- | --- | --- | --- | --- | --- | --- | --- |
| GQ | **** |  |  |  |  |  |  |  |
| WS | 2.0568 | **** |  |  |  |  |  |  |
| DHB | 1.4720 | 2.5384 | **** |  |  |  |  |  |
| YQ | 1.1145 | 2.7405 | 0.5806 | **** |  |  |  |  |
| SMX | 1.6490 | 2.1986 | 1.3671 | 0.9119 | **** |  |  |  |
| CFD | 1.7103 | 2.1062 | 1.2398 | 1.0709 | 1.4173 | **** |  |  |
| GD | 3.6258 | 1.5734 | 2.7769 | 2.5245 | 3.0546 | 2.7836 | **** |  |
| LD | 2.1296 | 2.0638 | 2.1844 | 2.5096 | 2.4525 | 2.1882 | 1.5929 | **** |

Note: The genetic distance matrix was calculated based on the Nei’s (1972) in PopGene32 software.

**Table S5** Primer sequences of 16S rRNA genes of bacteria and ITS1 regions of fungi

|  | Primer | Sequence 5’—3’ |
| --- | --- | --- |
| Bacteria | 515F | GTGCCAGCMGCCGCGG |
|  | 907R | CCGTCAATTCMTTTRAGTTT |
| Fungi | ITS1F | CTTGGTCATTTAGAGGAAGTAA |
|  | ITS2R | GCTGCGTTCTTCATCGATGC |

**Table S6** Significance test of foliar microbial community of α diversity index based on geographic location

|  | α diversity index | Geographic location | Mean | SE | P |
| --- | --- | --- | --- | --- | --- |
| Bacteria | Shannon | North | 2.63 | 0.56 | 0.27 |
|  |  | South | 2.12 | 1.10 |  |
|  | Richness | North | 174.41 | 109.54 | 0.41 |
|  |  | South | 159.63 | 136.98 |  |
| Fungi | Shannon | North | 2.83 | 0.62 | 0.13 |
|  |  | South | 2.11 | 1.11 |  |
|  | Richness | North | 341.17 | 124.92 | 0.04 |
|  |  | South | 240.88 | 93.96 |  |

Note: Mann-Whitney U test, P < 0.05.
